# Comparison between Focused Electron/Ion Beam-Induced Deposition at Room Temperature and under Cryogenic Conditions

**DOI:** 10.3390/mi10120799

**Published:** 2019-11-21

**Authors:** José María De Teresa, Pablo Orús, Rosa Córdoba, Patrick Philipp

**Affiliations:** 1 Instituto de Ciencia de Materiales de Aragón (ICMA, CSIC-Universidad de Zaragoza) and Departamento de Física de la Materia Condensada, Facultad de Ciencias, Universidad de Zaragoza, Calle Pedro Cerbuna 12, 50009 Zaragoza, Spain; porus@unizar.es; 2 Laboratorio de Microscopías Avanzadas (LMA), Instituto de Nanociencia de Aragón (INA), Edificio de I+D, Campus Río Ebro, 50018 Zaragoza, Spain; 3 Instituto de Ciencia Molecular, Universitat de València, Catedrático José Beltrán 2, 46980, Paterna, Spain; rosa.cordoba.castillo@gmail.com; 4 Advanced Instrumentation for Ion Nano-Analytics (AINA), MRT Department, Luxembourg Institute of Science and Technology (LIST), 41 rue du Brill, L-4422 Belvaux, Luxembourg; patrick.philipp@list.lu

**Keywords:** focused ion beam, focused electron beam-induced deposition, focused ion beam-induced deposition, lithography, circuit edit, electrical contacts, thin films, nanowires

## Abstract

In this contribution, we compare the performance of Focused Electron Beam-induced Deposition (FEBID) and Focused Ion Beam-induced Deposition (FIBID) at room temperature and under cryogenic conditions (the prefix “Cryo” is used here for cryogenic). Under cryogenic conditions, the precursor material condensates on the substrate, forming a layer that is several nm thick. Its subsequent exposure to a focused electron or ion beam and posterior heating to 50 °C reveals the deposit. Due to the extremely low charge dose required, Cryo-FEBID and Cryo-FIBID are found to excel in terms of growth rate, which is typically a few hundred/thousand times higher than room-temperature deposition. Cryo-FIBID using the W(CO)_6_ precursor has demonstrated the growth of metallic deposits, with resistivity not far from the corresponding deposits grown at room temperature. This paves the way for its application in circuit edit and the fast and direct growth of micro/nano-electrical contacts with decreased ion damage. The last part of the contribution is dedicated to the comparison of these techniques with other charge-based lithography techniques in terms of the charge dose required and process complexity. The comparison indicates that Cryo-FIBID is very competitive and shows great potential for future lithography developments.

## 1. Introduction

Lithography techniques are at the core of technological developments in fields such as nanoelectronics, data storage, sensors, telecommunication devices, quantum technologies, etc. [1]. Despite the fact that optical lithography is the most common technique for micro/nano-patterning, various niches exist for the application of other lithography techniques. In this context, charged-particle-based lithography techniques present some advantages compared to optical lithography, such as the affordable cost for sub-100-nm resolution and the capability for fast device prototyping. Amongst them, electron beam lithography (EBL) is the most popular, with patterning resolution down to a few nm using a scanning electron microscope (SEM) [2]. However, EBL is a multi-step process using electron-sensitive resists and requires long exposure times, which limits its applicability compared to optical lithography. Focused ion beam (FIB) is also a slow processing technique but is capable of direct material removal with a resolution of a few nm and without the need of resists [3,4]. This is why FIB milling has become the lithography of choice in various applications requiring local material removal, such as the circuit editing of semiconductor devices [5] and materials analysis in industrial and research laboratories for the preparation of cross-sections for scanning [6] and transmission electron microscopy [7].

## 2. Focused Electron/Ion Beam-Induced Deposition Techniques

If an SEM or a FIB is combined with a precursor gas, delivered by means of a gas injector and adsorbed on the substrate, precursor dissociation will occur after electron or ion impact on the surface, giving rise to the growth of a deposit. The corresponding techniques are known as FEBID (Focused Electron Beam-induced Deposition) and FIBID (Focused Ion Beam-induced Deposition), respectively. In the past, various review articles have been devoted to the detailed description of the phenomena involved in room-temperature (RT) FEBID and FIBID processes, and the reader is referred to these references for their comprehensive understanding [8,9,10,11,12,13]. Here, we provide a view that is simplified but sufficient to understand the main differences between the RT and the cryogenic processes.

In short, in FEBID, the process is commonly described through the time-variation of the precursor adsorbate density, which depends on four terms: adsorption, desorption, diffusion and dissociation. Each of these terms is characterized by the corresponding temperature-dependent coefficient. The growth volume can be obtained from the time integration of the dissociation term and the adsorbate density. Two extreme growth regimes can occur: in the electron-limited regime the growth rate depends linearly on the electron beam current or the electron dwell time, whereas, in the precursor-limited regime the growth rate saturates beyond a certain electron beam current or electron dwell time due to the lack of precursor replenishment in the area of growth. Microscopically, all types of electrons (primary, forward-scattered, back-scattered, type-I secondary and type-II secondary) can potentially contribute to the precursor dissociation depending on the working conditions, as previously discussed in great detail in references 8 to 13 and others [14,15,16,17]. In general, the primary, forward-scattered and back-scattered electrons only dissociate a reduced amount of precursor molecules given the large difference in energy of the impacting electron (up to tens of keV) and the precursor molecule bond energies (a few eV). A greater contribution to precursor dissociation comes from type-I and type-II secondary electrons, produced in the substrate and in the growing structure. The secondary electrons, with typical energies in the range of a few eV, have a higher probability of precursor bond-breaking. Temperature plays a fundamental role, owing to the large thermal dependence of important process parameters such as the diffusion coefficient and the residence time of the precursor on the surface.

On the other hand, in FIBID, the material growth has to compete with FIB milling, with both phenomena occurring simultaneously [18]. As a consequence, in the first approximation, the growth rate is given by the subtraction of the milling rate from the precursor dissociation rate. FIB milling can be reduced by the use of low ion beam currents [19]. An advantage of FIBID compared to FEBID is that each impacting ion triggers physical processes on the substrate that produce a large amount of secondary electrons on the substrate surface. As a consequence, for a given beam current and accelerating voltage, the growth rate is typically a factor of 100 higher in FIBID compared to FEBID [20]. For the growth of metallic deposits, FIBID will generally give rise to higher metal content and lower resistivity [20], which is an obvious advantage in certain applications. However, in sharp contrast with FEBID, FIBID causes substantial beam-induced damage on the substrate (amorphization, implantation, defects, etc.), which can be detrimental in some cases [21,22,23,24].

**Figure 1:**
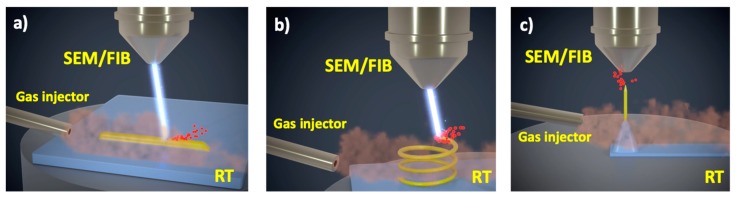
Three different applications of Focused Electron Beam-Induced Deposition (FEBID) and Focused Ion Beam-induced Deposition (FIBID) growth are sketched: (**a**) In-plane nanowires on flat substrates; (**b**) Three-dimensional nanostructures; (**c**) Nanowire growth on tips and cantilevers. RT stands for room temperature.

In Figure 1, various applications of FEBID and FIBID are sketched: the growth of in-plane and three-dimensional nanostructures on flat substrates [25], as well as on unconventional substrates such as cantilevers/tips [26], and flexible [27], insulating [28] or origami substrates [28], etc. Materials grown by FEBID/FIBID are currently used for circuit edit and mask repair in the semiconductor industry [24,29,30,31], lamellae preparation [7], the placement of electrical contacts to micro- and nano-structures [32,33], for producing sensors [34,35] and magnetic tips [36,37,38], plasmonic [39,40,41,42] and nano-optical elements [43], superconducting films [44] and nanowires [45], etc. Although FEBID/FIBID is an active field of research and development, a wider impact is hampered by the limited process speed. For example, the growth of Pt-C deposits (the most popular one) by FEBID, using the (CH_3_)_3_Pt(C_p_CH_3_) precursor, shows a volume per dose of 0.005 μm^3^/nC under 30 kV, increasing to around 0.7 μm^3^/nC for Pt-C deposits by FIBID [20]. This amounts to long processing times, especially for FEBID. As an example, a 10 μm x 10 μm Pt-C square of 0.1 μm thickness (total volume of 10 μm^3^) grown using 1 nA beam current requires 14 s using FIBID and more than half an hour using FEBID. The situation is even worse with other popular precursors such as W(CO)_6_, with values of volume per dose that are several orders of magnitude smaller than for the Pt precursor.

## 3. Focused Electron/Ion Beam-Induced Deposition Techniques under Cryogenic Conditions (Cryo-FEBID and Cryo-FIBID)

In the recent past, various lithography processes under cryogenic conditions have been developed. For example, processes based on water ice and organic ice have been carried out [46,47,48]. Here, the ice plays the role of the resist in Electron Beam Lithography (EBL), with the advantage that the unexposed ice becomes volatile when heating up to room temperature, instead of requiring a resist solver as in EBL. So far, the main bottleneck of this technology is the high charge dose required to expose the ice, as well as the need for subsequent steps, such as metal coating and lift-off for the fabrication of a functional nano-patterned material. On the other hand, during FIB milling, cryogenic temperatures have been found useful in order to retain the microstructure of sensitive materials for subsequent scanning and transmission electron microscopy imaging [49,50]. Also, in gas-assisted focused electron beam-induced etching, cryogenic temperatures (Cryo-FEBIE) have been found to increase the etching rate by enhancing the residence time of the etching gas [51]. Regarding deposition techniques, thin Sn films were grown under cryogenic temperatures using a condensed layer of an Sn precursor after exposure to a broad Ar^+^ beam, but good lateral resolution was not attempted [52].

**Figure 2:**
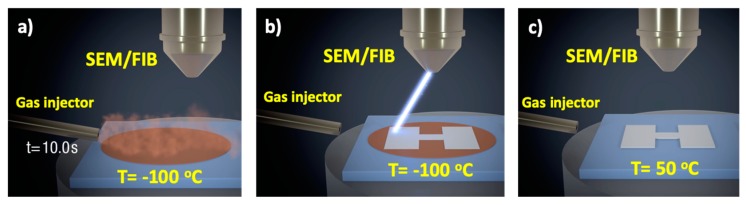
The three main steps of FEBID and FIBID under cryogenic conditions are sketched: (**a**) The precursor is dosed for a given time on a cooled substrate, giving rise to a condensed layer of precursor; (**b**) The focused electron or ion beam irradiates the precursor condensed layer with the wanted pattern; (**c**) The substrate is heated to above room temperature, which produces the sublimation of the unirradiated precursor and the emergence of the desired deposit.

The first example of direct growth of a nanopatterned material by cryogenic focused beam-induced deposition techniques was reported by Bresin et al. using the (CH_3_)_3_Pt(C_p_CH_3_) precursor and electron irradiation, known as Cryo-FEBID [53,54]. The second example was reported by Córdoba et al. using the W(CO)_6_ precursor and ion irradiation, known as Cryo-FIBID [55]. Figure 2 illustrates the steps involved in such a process. The first step involves cooling the substrate below the condensation temperature of the precursor and opening the Gas Injection System (GIS) for a given time to allow the formation of a precursor condensation layer. The thickness of such a condensed layer is very important, as will be discussed later, and can be controlled by several means, such as the GIS valve opening time, the precursor temperature inside the GIS, and the distance between the GIS and the substrate. The second step implies the focused beam irradiation, with the obvious advantage over the RT process that the required irradiation dose is much smaller. As the condensed layer contains many precursor molecules available for dissociation compared to the sub-monolayer precursor layer at RT, the irradiation process is much faster. The parameters to be controlled during the irradiation process are the usual ones in FEBID/FIBID: beam current, beam voltage, scan type, dwell time, pitch, etc. Once the optimal irradiation dose has been determined, the next step to control is the heating. In this step, the non-irradiated part of the condensed layer will become volatile and only the irradiated part of the condensed layer will remain. Heating to temperatures slightly above RT, for example at 50 °C for a few minutes, has been found suitable in this step. Then the sample can be brought back to RT for further processing.

### 3.1. Pt-C Deposits Grown by Cryo-FEBID

**Figure 3:**
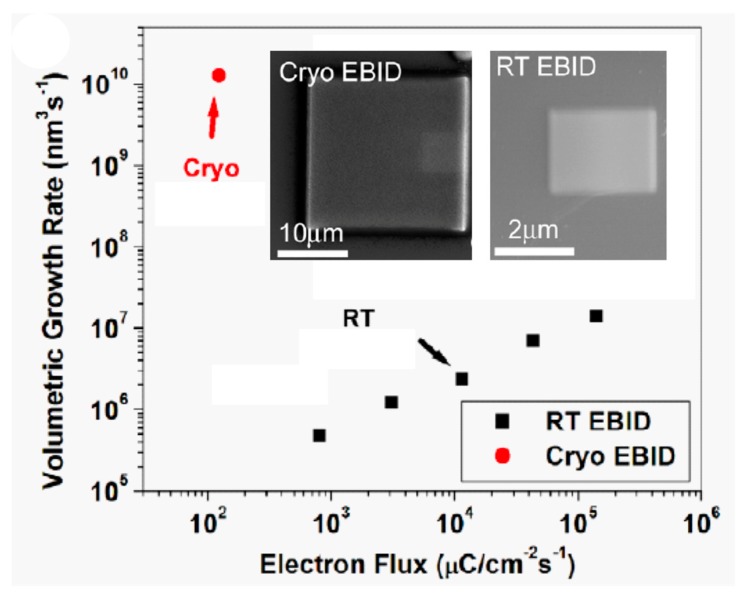
Growth rate enhancement of Pt-C deposits grown by Cryo-FEBID compared to those grown using RT FEBID. The inset shows SEM micrographs of the obtained deposits. Adapted and reprinted from Bresin et al., Nanotechnology 2013 [54].

Bresin et al. have used layers of (CH_3_)_3_Pt(C_p_CH_3_) precursor condensed at −155 °C, with focused electron irradiation under 15 kV beam energy and 0.71 nA beam current [54]. As shown in Figure 3, the main result is an increase of four orders of magnitude in the growth rate by Cryo-FEBID, reaching values in the range of 10^3^ C/cm^2^, similar to electron doses required for the exposure of resists in EBL with PMMA resist [2]. The condensate thickness was varied by Bresin et al. in the 75 nm to 300 nm range by tuning the GIS temperature, the GIS-substrate distance and the GIS valve opening time. The authors report that the Pt content (in at %) is 14%, similar to that obtained when using RT FEBID. The authors also showed strategies to achieve 3D structures by tailored multiple precursor-layer condensation and irradiation. In short, the results obtained were promising for the fast growth of Pt-C structures by Cryo-FEBID. However, no particular application was discussed or targeted.

### 3.2. W-C Deposits Grown by Cryo-FIBID

**Figure 4:**
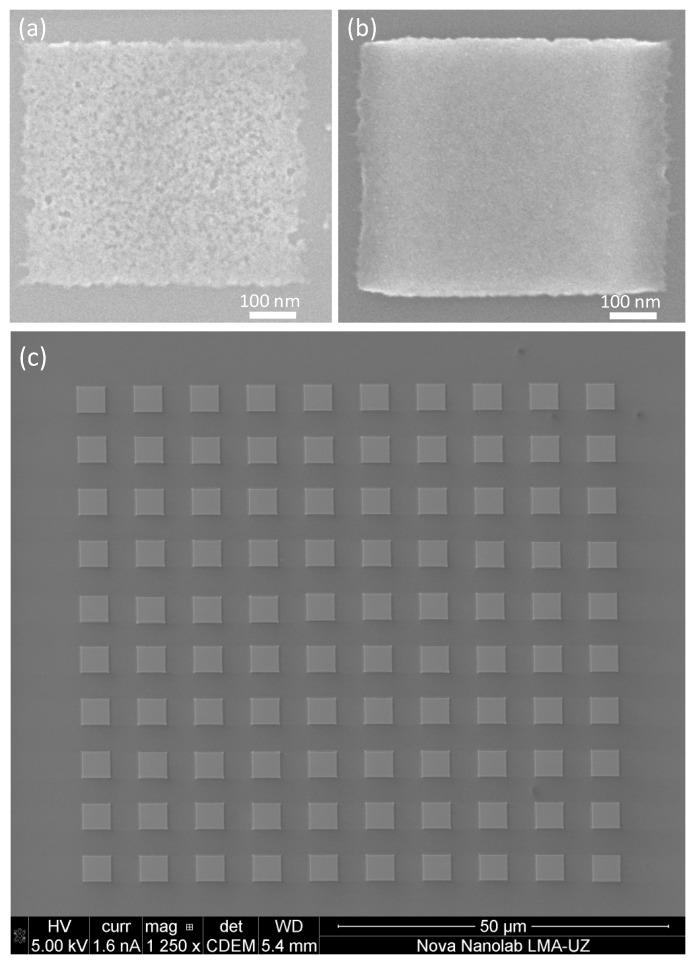
(**a**) SEM micrograph of a W-C 30-nm thick Cryo-deposit irradiated with a 4.21 μC/cm^2^ dose (unoptimized dose); (**b**) SEM micrograph of a W-C 30-nm thick Cryo-deposit irradiated with a 35.7 μC/cm^2^ dose (optimized dose); (**c**) SEM micrograph of a W-C Cryo-deposit array, composed of 100 rectangles of 4 μm^2^ x 3.85 μm^2^ in size, grown in a single Ga^+^ irradiation exposure using an irradiation dose of 50 μC/cm^2^, amounting to a total irradiation time of 85 s (compared with 14 h using RT FIBID). Adapted and reprinted from Córdoba et al., Scientific Reports 2019 [55].

Córdoba et al. have recently used 30-nm thick layers of W(CO)_6_ precursor condensed at −100 °C, with focused Ga^+^ ion irradiation under 30 kV beam energy and 10 pA beam current [55]. As shown in Figure 4, such working conditions plus ion doses in the range of 50 μC/cm^2^ have been found favorable for obtaining homogeneous void-free W-C deposits. The edge roughness of deposits grown with the optimized dose, of the order of 10 nm, can be related to the ion beam size as well as to mechanical instabilities and drifts caused by various noise sources, including the vibrations coming from the flow of the gaseous nitrogen required to cool the stage. The required ion dose is 600 times lower than that required to obtain equivalent deposits at RT FIBID. Such ultra-fast growth is of great interest for decreasing the process time. As an example, the 100 micrometric rectangles shown in Figure 4b only required 85 s of Ga^+^ irradiation, whereas the equivalent deposit grown by RT FIBID would take 14 h.

All the compositional analyses of the W-C Cryo-deposits, performed by EDX-STEM techniques, indicated a gradient of composition as a function of the deposit height. The amount of W is above 20% in the top half of the deposit, close to the surface, but decreases to below 10% in the bottom half of the deposit, near the substrate. In order to understand this behavior, simulations on 30 kV Ga^+^ irradiation at a normal incidence of a 30 nm thick W(CO)_6_ film on a silicon substrate have been performed using the SDTRIMSP code [56], which is based on TRIM [57,58] but allows for dynamics simulations, modelling ion-beam processes as a function of fluence while taking diffusion processes into account [59,60]. For the simulations in this work, the KrC potential has been used for interatomic interactions, the Oen-Robinson model for electronic stopping, and the Gauss-Mehler method with 16 pivots for integration. The surface binding energy is calculated using $sbe\left(i,j\right)=0.5\left(Es_{i}+Es_{j}\right)$, where sbe is the surface binding energy for the target of consideration, and $Es_{i}$ is the atomic surface binding energy and where the surface binding energy of atom *i* is calculated for any combination of Ga, C, O, W and Si [56]. The atomic densities of tungsten, carbon and oxygen have been considered identical to the bulk values, which may lead to a density of the precursor film above the experimental value. The diffusion of atoms has also been neglected as no experimental values were available for the experimental conditions of the current work. However, this process could be relevant for the diffusion of carbon and oxygen atoms under Ga irradiation in Cryo-FIBID, meaning that the surface concentrations of oxygen and carbon in the simulations might be overestimated and that of tungsten underestimated. In a previous study on the rare gas ion irradiation of polymer samples, including diffusion processes was essential for a correct modelling of the processes under ion irradiation [60,61].

**Figure 5:**
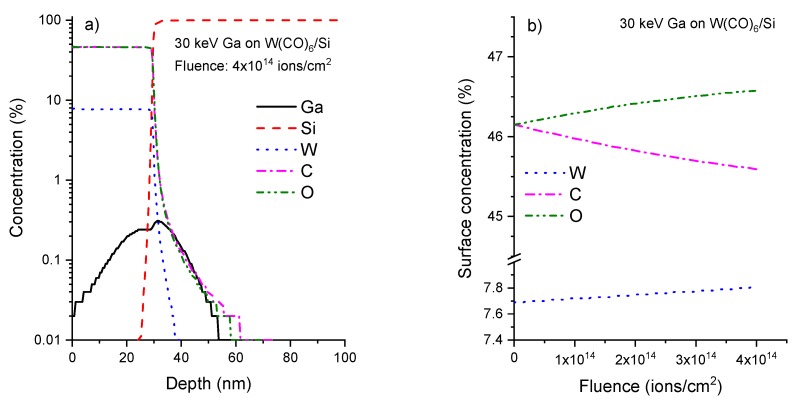
(**a**) Theoretical composition as a function of depth for Cryo-FIBID W-C deposits according to the calculations reported in the main text for a fluence of 4 × 10^14^ ions/cm^2^; (**b**) Surface composition as a function of fluence for the same modelling conditions as for (**a**).

**Figure A1:**
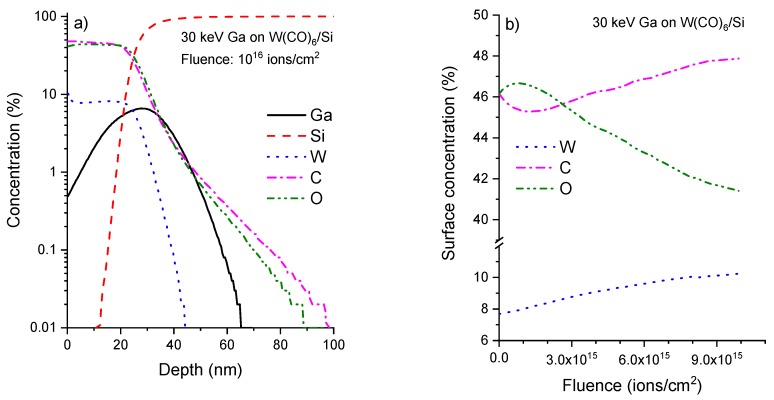
(**a**) Theoretical composition as a function of depth for Cryo-FIBID W-C deposits according to the calculations reported in the main text for a fluence of 1 × 10^16^ ions/cm^2^; (**b**) Surface composition as a function of fluence for the same modelling conditions than for (**a**).

In the current work, the simulated tungsten concentration of about 8% at a fluence of 4 × 10^14^ ions/cm^2^ (Figure 5), which is in the range of the optimized irradiation dose according to Córdoba et al. [55], is also lower than the experimental surface concentration of above 20%, which gives an indication that diffusion processes and/or outgassing after the dissociation of the precursor molecules play a role but are not accounted for in the modelling. Although the W surface concentration starts to increase in the simulations with a fluence of 4 × 10^14^ ions/cm^2^ (Figure 5b), the tungsten surface concentration only starts to be significantly above the initial concentration of the precursor film for fluences above 10^16^ ions/cm^2^ (see Figure A1 in Appendix A). For the fluence corresponding to experimental conditions, the Ga surface concentration is still low, with the maximum of the implantation profile being more or less at the W(CO)_6_/Si interface (Figure 5a). For higher fluences in the range of 10^16^ ions/cm^2^, the Ga concentration starts approaching the percent range at the sample surface and a peak concentration of about 10% range (see Appendix A). For the lower fluences, the loss of carbon is dominant while at higher fluences the loss of oxygen prevails. Partial sputtering yields are almost constant, with 0.06 for W, 0.92 for C and 1.16 for O.

**Figure 6:**
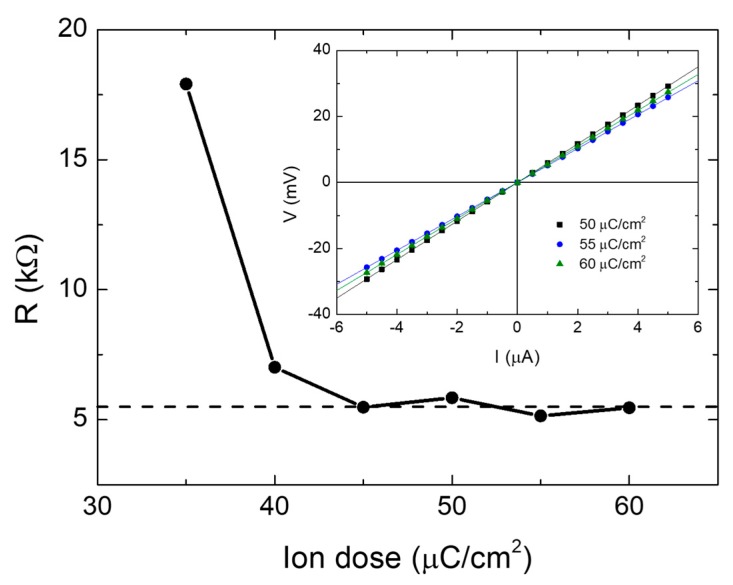
Electrical resistance as a function of the Ga^+^ irradiation dose in W-C deposits grown by Cryo-FIBID, suggesting that an optimized dose to achieve the lowest resistance value occurs in the 45 to 60 μC/cm^2^ range. The current-versus-voltage measurements shown in the inset are compatible with metallic behavior. Adapted and reprinted from Córdoba et al., Scientific Reports 2019 [55].

Since the main application of W-C FIBID deposits is based on their metallic character, it is important to investigate whether the W-C deposits grown by Cryo-FIBID exhibit it. As shown in Figure 6, these deposits display metal behavior and can be used as metallic interconnects. The observed resistivity, 800 μΩcm, is slightly higher than that in the corresponding W-C deposits grown by RT FIBID, 300 μΩcm, which is to be expected due to the larger metal content in the latter [62,63]. The value of 300 μΩcm for room-temperature FIBID is just an average of results found in the literature. However, depending on the growth parameters, the deposit thickness, the vacuum conditions or the state of the precursor material, amongst others, it ranges from 100 μΩcm to 3000 μΩcm [27,62,64,65,66]. Similarly, the resistivity of other conductive FIBID deposits, such as Pt-C, also spans a broad range (from 700 μΩcm to 10^8^ μΩcm) given its strong dependence on the deposit thickness [67]. On the other hand, W-C deposits grown by RT FEBID show resistivity values at least ten times higher than RT FIBID [68].

Regarding the lateral resolution of Cryo-FEBID, Bresin et al. showed that 40 nm is achievable with a 70-nm thick condensed layer [54]. The lateral resolution worsens to 140 nm if a 280-nm thick condensed layer is used. On the other hand, Córdoba et al. have shown a 38-nm lateral resolution in Cryo-FIBID with a 30-nm thick condensed layer [55]. The proximity effect observed in that work is surprisingly small, with two nanowires separated by a 7 nm gap. The application of Cryo-FIBID for the fabrication of highly-dense nano-objects is thus very promising.

It is also worth mentioning that Cryo-FEBID and Cryo-FIBID processes can be repeated multiple times on the same sample in order to produce 3D structures, or to grow various materials or to increase the thickness of the deposited material, widening the range of applications [54].

## 4. Discussion and Conclusions

The use of cryogenic conditions implies the integration of a cryogenic module in the equipment, spending a few s or minutes lowering (and subsequently increasing) the temperature and coping with effects, such as temperature-induced drifts in the mechanical parts of the equipment. The outstanding time saving of FEBID and FIBID processes with condensed precursor layers is one benefit that outweighs the inconveniences of working under cryogenic conditions. In the case of Pt-Cryo-FEBID, the growth rate has been found to increase by about four orders of magnitude compared to the equivalent RT process [54], whereas in the case of W-Cryo-FIBID, it is enhanced about 600 times [55]. This will definitely have a great impact in the total process time when large areas need to be patterned, as shown in Figure 4.

**Figure 7:**
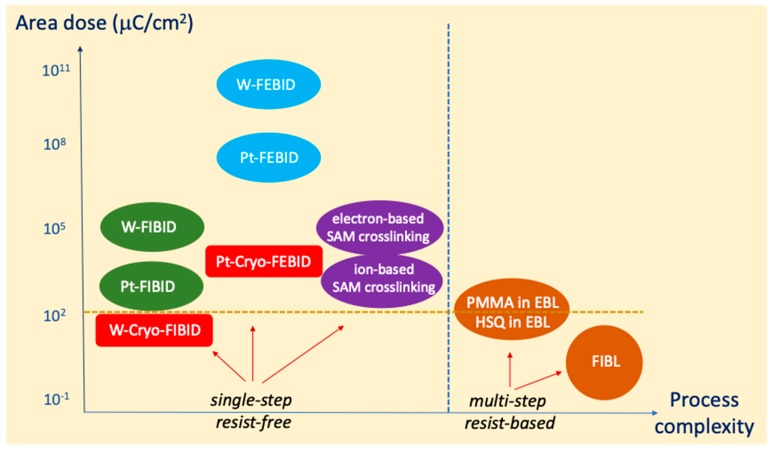
Comparison of charge-particle-based lithography techniques in terms of the required charge dose per area and the process complexity. The single-step or multi-step character of the technique is considered a means to classify it as a process with less or more complexity, respectively. Cryo-FIBID requires the lowest charge dose amongst the single-step techniques.

In order to put the obtained results in context, the area dose required in the main charged-particle-based nanolithography techniques is displayed in Figure 7. Such lithography techniques can be classified depending on whether they are single-step resist-free or multi-step resist-based. The most common resist-based nanolithography technique using charge particles is EBL. Processes based on PMMA resist are widespread due to the achievement of high resolution requiring moderate electron doses, in the range of a few-hundred μC/cm^2^ [2]. Faster EBL processes, with doses lower than 100 μC/cm^2^, are possible using specific resists such as HSQ (hydrogen silsesquioxane) [2]. Even faster resist-based processes (a few μC/cm^2^) have been described by Focused Ion Beam Lithography (FIBL) using PMMA, HSQ or fullerene-based resists with He^+^ irradiation exposure [69,70,71] and SAL 601 resist with Ga^+^ irradiation exposure [72]. After resist exposure, a few more steps have to be followed, typically resist development, metal evaporation and lift-off, which leads to long processing times. In some cases, resist residues are detrimental and additional steps are required. However, single-step resist-free nanolithography processes have the advantage of being simple if the right material can be produced. In fact, great effort has been made in recent years to obtain functional materials by FEBID and FIBID, which can include a purification step [73,74]. In most FEBID and FIBID processes, the required charge dose is high, giving rise to long processing times with the exception of Pt deposits by FIBID, which require an irradiation dose of around 10^3^ μC/cm^2^ [20]. The use of FIBID implies ion implantation and ion-induced damage, which can be detrimental in certain applications. Thus, the use of FEBID and FIBID at room temperature presents limitations in speed or causes damage that hampers a broader use. In Figure 7, another example of a single-step resist-free charge-based nanolithography technique is included, that of the formation of ultra-thin carbon membranes from the exposure of self-assembled monolayers (SAM), which require at least 850 μC/cm^2^ [75]. As shown in Figure 7, the use of Cryo-FEBID and Cryo-FIBID opens new opportunities due to the lower area charge dose required. In the case of Cryo-FEBID, if this technique is able to demonstrate material functionality, it can significantly decrease the processing times of RT FEBID. In the case of Cryo-FIBID, the two main problems linked to RT FIBID can be minimized. First, the processing time is substantially decreased compared to the rest of existing single-step resist-free methods, given the required low dose of 50 μC/cm^2^ [55]. Secondly, the ion implantation and ion-induced damage can to a large extent be suppressed. Cryo-FIBID has shown material functionality, leading to metallic W-C deposits used to contact a semiconducting nanowire for the investigation of its electrical properties [55].

Following the previous discussion, it is fair to anticipate the potential applications of Cryo-FEBID and Cryo-FIBID. In the field of circuit edit, Cryo-FIBID seems a promising technique to replace RT FIBID given the existing demonstration of metallic W-C Cryo-deposits requiring a very low charge dose [55]. For mask repair, both, Cryo-FEBID and Cryo-FIBID, could play a relevant role in decreasing the processing time if the right material deposition is demonstrated in the future. Besides, Cryo-FEBID has been shown to be a viable technique that would span the range of etching processes available [51]. However, we would like to point out that cryogenic processes bring about unavoidable thermal drifts, which could limit the implementation of the technique in the most demanding high-resolution applications.

Moreover, Cryo-FIBID can be used straightforwardly to place metallic contacts for the investigation of the electrical properties of nano-objects with minimized ion-induced damage. Thus, one can anticipate that research of 2D, epitaxial oxides, very-thin nanowires and topological insulators could benefit from it. It could even be applied to other materials such as organic layers if these can maintain their properties when submitted to the cooling and heating process. More generally, given that the low charge doses required in Cryo-FEBID and Cryo-FIBID are comparable to those used in multi-step resist-based processes, it is not unreasonable to consider these techniques for large-scale nanopatterning processes. Thus, the growth of large-scale metal contacts on resist-sensitive materials, or the growth of large-scale unique functional materials by FEBID and FIBID, or the growth of large-scale nanoscale hard masks, can be obtained from these techniques. For example, large-area patterning, in the cm^2^ range, has been developed for EBL. For that, advanced stages relying on laser interferometry are used to optimize stitching. Nevertheless, given that Cryo-FIBID requires a homogeneous condensed layer, new designs of gas injection systems would prove useful for precursor delivery in large areas.

Furthermore, the use of other FIB sources different from Ga^+^ (such as He^+^, Ne^+^, Xe^+^, etc.) provides another opportunity to enlarge the applicability of the Cryo-FIBID technique [3]. Depending on the type of ion and its accelerating voltage and working current, access to a wide range of irradiation depth, resolution, process time, and material functionality exists.

To sum up, in the present article we compare the performance of focused beam-induced deposition techniques under cryogenic temperatures compared to conventional room-temperature processes. With required area charge doses a few orders of magnitude lower than conventional RT FEBID and FIBID, Cryo-FEBID and Cryo-FIBID show the main advantage of the gain in process speed. Cryo-FIBID is found to be very promising, given the demonstration of metallic W-C deposits with an irradiation dose of around 50 μC/cm^2^. These low ion doses pave the way for the application of Cryo-FIBID on ion-sensitive materials, which usually become damaged with the conventional irradiation doses used in RT FIBID. Other nanopatterning strategies based on Cryo-FEBID and Cryo-FIBID, and competitive with respect to well-established multi-step resist-based approaches, are expected to come in the future.
