# Erratum: De Teresa, J.M. et al. Comparison between Focused Electron/Ion Beam-Induced Deposition at Room Temperature and under Cryogenic Conditions. *Micromachines* 2019, *10*, 799

**DOI:** 10.3390/mi11020211

**Published:** 2020-02-18

**Authors:** José María De Teresa, Pablo Orús, Rosa Córdoba, Patrick Philipp

**Affiliations:** 1 Instituto de Ciencia de Materiales de Aragón (ICMA, CSIC-Universidad de Zaragoza) and Departamento de Física de la Materia Condensada, Facultad de Ciencias, Universidad de Zaragoza, Calle Pedro Cerbuna 12, 50009 Zaragoza, Spain; porus@unizar.es; 2 Laboratorio de Microscopías Avanzadas (LMA), Instituto de Nanociencia de Aragón (INA), Edificio de I+D, Campus Río Ebro, 50018 Zaragoza, Spain; 3 Instituto de Ciencia Molecular, Universitat de València, Catedrático José Beltrán 2, 46980 Paterna, Spain; rosa.cordoba.castillo@gmail.com; 4 Advanced Instrumentation for Ion Nano-Analytics (AINA), MRT Department, Luxembourg Institute of Science and Technology (LIST), 41 rue du Brill, L-4422 Belvaux, Luxembourg; patrick.philipp@list.lu

In Section 3.1 (page 4) [1], on the fourth line, it says “C/cm^2^”. It should be changed to “μC/cm^2^”. 

The editorial office would like to apologize for any inconvenience caused to the authors and readers.
